# Integrating Mechanistic and Toxicokinetic Information in Predictive Models of Cholestasis

**DOI:** 10.1021/acs.jcim.3c00945

**Published:** 2023-09-03

**Authors:** Pablo Rodríguez-Belenguer, Victor Mangas-Sanjuan, Emilio Soria-Olivas, Manuel Pastor

**Affiliations:** † Research Programme on Biomedical Informatics (GRIB), Department of Medicine and Life Sciences, Universitat Pompeu Fabra, Hospital del Mar Medical Research Institute, 08003 Barcelona, Spain; ‡ Department of Pharmacy and Pharmaceutical Technology and Parasitology, Universitat de València, 46100 Valencia, Spain; § Interuniversity Research Institute for Molecular Recognition and Technological Development, Universitat Politècnica de València, 46100 Valencia, Spain; ∥ IDAL, Intelligent Data Analysis Laboratory, ETSE, Universitat de València, 46100 Valencia, Spain

## Abstract

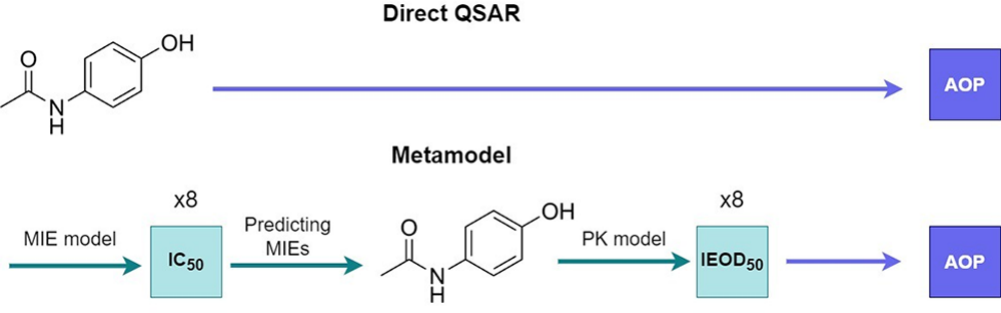

Drug development involves the thorough assessment of the candidate’s safety and efficacy. *In silico* toxicology (IST) methods can contribute to the assessment, complementing *in vitro* and *in vivo* experimental methods, since they have many advantages in terms of cost and time. Also, they are less demanding concerning the requirements of product and experimental animals. One of these methods, Quantitative Structure–Activity Relationships (QSAR), has been proven successful in predicting simple toxicity end points but has more difficulties in predicting end points involving more complex phenomena. We hypothesize that QSAR models can produce better predictions of these end points by combining multiple QSAR models describing simpler biological phenomena and incorporating pharmacokinetic (PK) information, using quantitative *in vitro* to *in vivo* extrapolation (QIVIVE) models. In this study, we applied our methodology to the prediction of cholestasis and compared it with direct QSAR models. Our results show a clear increase in sensitivity. The predictive quality of the models was further assessed to mimic realistic conditions where the query compounds show low similarity with the training series. Again, our methodology shows clear advantages over direct QSAR models in these situations. We conclude that the proposed methodology could improve existing methodologies and could be suitable for being applied to other toxicity end points.

## Introduction

There is an urgent need to replace, reduce, and refine (3Rs) animal experimentation. The knowledge obtained from past *in vivo* experiments can be reused to minimize the need to perform new assays, promoting sustainable science. New approach methodologies (NAMs) constitute an attractive alternative for assessing chemical hazards and estimating the effects of exposure, with the potential to support Toxicological Next Generation Risk Assessment (NGRA) and to promote the application of the 3R principles. Among the different approximations encompassed by the NAM term,^1,2^*in silico* methods are highly convenient on their own or as a complement to *in vitro* techniques.

While *in silico* toxicology (IST) offers benefits in terms of cost-effectiveness, high throughput, and ethical considerations, its ability to predict complex biological end points is still under debate.^3^ Another difficulty is its integration with experimental data for risk assessment purposes, particularly in regulatory setups.^4−7^

Quantitative structure–activity relationship (QSAR) is one of the most used methodologies in the IST field. It has been successfully used to predict *in vitro* results and simple toxicological end points.^8,9^ However, the predictivity of QSAR models becomes limited when it comes to complex biological end points, such as organ toxicity. This is because complex biological end points result from multiple mechanisms and effects at different biological levels, making it more challenging to predict them accurately with QSAR. Additionally, QSAR models have only local validity, and the low structural similarity between the compounds in the validation and training sets can result in poor predictive performance.^10^ Another drawback of the QSAR models is that, usually, they do not consider pharmacokinetic (PK) information, such as the absorption, distribution, metabolism, and excretion (ADME) properties of compounds,^11^ and they might have difficulties in characterizing the actual chemical risk of a compound since the toxicity of a compound is linked to the exposure.^12^

QSAR methods can be important in transitioning to mechanism-based toxicology.^13^ In this quest, Adverse Outcome Pathways (AOPs) have been developed to integrate existing mechanistic knowledge into a rational framework.^14^ An AOP connects known biological events linearly through a series of Key Events (KEs) from a Molecular Initiating Event (MIE) to the final Adverse Outcome (AO). The causal relationships between these KEs are defined by Key Event Relationships (KERs).

In 2013, the Organization for Economic Co-operation and Development (OECD) published the first version of the Guidance Document on Developing and Assessing Adverse Outcome Pathways with a conceptual background,^15^ followed by the publication of the User’s Handbook Supplement in 2018.^16^ This supplement provides practical guidance and advice on applying AOPs in the context of risk assessment and highlights the benefits of using a mechanistic approach to comprehend adverse effects better. Moreover, this supplement contains practical instructions for AOP development and collaborative work on the databases AOP knowledgebase (AOP-KB)^17^ and AOP-Wiki.^18^

Computational methods could exploit the standardized knowledge representation that AOPs provide. Accordingly, *in silico* models with multiple molecular initiating events (MIEs) can be built to predict complex toxicological end points for which QSAR models do not provide quality results.^19,20^

AOPs have also been incorporated in mechanistic-based toxicokinetic (TK)/toxicodynamic models that evaluate exposure–response relationships.^21−23^ A common misconception is to consider that drugs with a very small IC_50_ are “more toxic”. However, this is not necessarily true, as the likelihood and severity of adverse effects are more closely linked to the total amount of drug at the target site, rather than the drug’s potency.^24^ Even drugs with a high IC_50_ can cause toxicity if the dose administered in clinical use (the therapeutic dose [T_D_]) is high enough. Therefore, to make decisions about the potential toxicity of drugs, IC_50_ values should be transformed to “point of departure doses” using quantitative *in vitro* to *in vivo* extrapolation (QIVIVE) models.^25^ QIVIVE is derived from a minimal physiological-based pharmacokinetic (PBPK) model, which reproduces the kinetics of a substance within a living organism over time, considering the main pharmacokinetic phenomena: absorption, distribution, metabolism, and excretion. The main objective of QIVIVE is to establish the *in vivo* dose which will produce a certain concentration in the blood (or tissues). This can correspond to *in vitro* concentrations such as the half-maximal effective concentration (EC_50_), IC_50_, or half-maximal active concentration (AC_50_). In this sense, QIVIVE can be considered a “reverse dosimetry” method, providing doses from concentrations.

In this work, we aim to develop a novel approach that integrates the contribution of multiple MIEs and the compound TK properties for the prediction of a complex toxicological end point. In this study, we will use hepatotoxicity as a representative example of a complex toxicological end point. Drug-induced liver injury (DILI) is one of the primary causes of attrition during clinical and preclinical studies and one of the main reasons for drug withdrawal from the market.^26,27^ DILI can be categorized as either idiosyncratic or nonidiosyncratic based on its relationship with the drug dose. If DILI occurs independently of the dose, it is considered idiosyncratic, while it is considered nonidiosyncratic if DILI is dose-dependent. Nonidiosyncratic DILI can be classified into the following three categories:^26^ (i) hepatocellular, (ii) cholestatic, and (iii) mixed. Because DILI is a very broad end point, we will focus on cholestasis.

Cholestatic DILI is a dose-dependent adverse effect defined as a disruption of the bile flow, which increases hepatic bile acid concentrations, resulting in necrosis and/or apoptosis. Together with the hepatocellular effect, it is one of the most severe manifestations of DILI.^26,28^ Cholestasis is often produced by inhibiting the hepatic transporters responsible for facilitating bile flow from the liver to the small intestine.^26^ Hepatic transporters are classified according to their location in the membranes: those belonging to the canalicular membrane and those belonging to the basolateral membrane. Canalicular membrane transporters regulate hepatic clearance, as well as the secretion of bile salts and conjugates into the bile. Basolateral membrane transporters regulate the uptake of drugs and transport endobiotics and xenobiotics from the blood to the hepatocyte.^26^

Bile Salt Export Pump (BSEP), multidrug resistance-associated protein (MRP2), Breast cancer resistance protein (BCRP), and P-glycoprotein (P-gp) are canalicular membrane transporters,^28−30^ while MRP3, MRP4, and organic anion transporting polypeptides (OATP1B1 and OATP1B3) are basolateral membrane transporters. The role of BSEP inhibition is one of the most important mechanisms studied in cholestasis occurrence, being the main MIE described in the cholestasis AOP found in the AOP-Wiki.^31^

This study aims to add to existing QSAR methodologies a new approach which integrates mechanistic information for multiple MIEs (using AOPs) and TK information (using QIVIVE models), providing a more complete and realistic description of the phenomenon studied. This approach is illustrated by applying it to the prediction of the cholestatic properties of a series of compounds. The results of this case study will be used to discuss its advantages compared to direct QSAR modeling, especially in the most common situations in drug development, where the candidates do not have much structural resemblance with the structures in the training series.

## Materials and Methods

### Cholestasis Data Set

A series of chemical compounds with cholestasis annotations was obtained from Kotsampasakou and Ecker (2017),^27^ where the researchers extracted the annotations from PubMed (http://www.ncbi.nlm.nih.gov/pubmed), Google, Scopus (https://www.scopus.com/), and the SIDER database v2 by searching the terms: “drug-induced cholestasis” and “cholestasis”. The data were curated by removing inorganic compounds and compounds containing metallic elements. In the end, the series consisted of 577 compounds with 130 “positives” (cholestatic compounds) and 447 “negatives” (noncholestatic compounds).

For our study, we applied additional curation, eliminating compounds whose administration route is neither oral nor intravenous, since only these routes provide relatively simple and well-understood absorption and elimination pathways. The filtered data set contained 437 compounds (116 positives and 321 negatives).

The compounds in this series were characterized using unique IDs to facilitate the extraction of data from other sources: ChEMBL IDs were obtained using chembl-webresource-client 0.10.8,^32^ DSSTox substance IDs or DTXSIDs were assigned using PubChemPy 1.0.4,^33^ and Drugbank IDs were obtained from Drugbank version 5.1.9.^34^ In addition, for chemical comparisons by pharmacological groups, information on Anatomical Therapeutic Chemical (ATC) classification was added up to the second level of information (Pharmacological or Therapeutic subgroup) using chembl-webresource-client and MedCode 1.3.^35^

### Transporter QSAR Models

Sets of compounds annotated with the pIC_50_ values were extracted from ChEMBL version 29 to build QSAR models for the main hepatic transporters involved in drug-induced cholestasis (low-level models, LLMs). The process of selecting compounds that inhibit specific transporters involved two filters: the target organism (*Homo sapiens*) and the target type (single protein). No filtering based on assay type was implemented, to avoid compromising the number of compounds selected. This decision was made taking into account that a higher number of assays might introduce variability due to differences in experimental conditions and measurement techniques.^36^ Compounds for which multiple experimental annotations were available were included as multiple data points. This procedure has the advantage of giving more weight to multiple-tested compounds and incorporating experimental variability, in contrast to alternative procedures in which a single mean or median is used to characterize their biological properties. The structures were standardized using a curation tool,^37^ removing inorganic compounds and compounds with metallic elements. Table 1 shows the hepatic transporters considered, with detailed information (transporter names with their target ChEMBL ID, acronym, number of compounds, and the mean and standard deviation [std] of the pIC_50_ distributions for each selected transporter) on the data extracted. To match the transporter inhibition data with the *in vivo* cholestasis data described above, we used the ChEMBL ID of the compounds.

**Table 1:** Information about Compounds Collected for Each Hepatic Transporter

**Transporter**	**Acronym**	***N***^a^	**Mean_pIC**_**50**_	**Std_pIC**_**50**_
P-glycoprotein (CHEMBL4302)	P-gp	1031	5.89	1.11
Breast cancer resistance protein (CHEMBL5393)	BCRP	1015	5.99	0.75
Organic anion transporting polypeptide 1 (CHEMBL1697668)	OATP1B1	63	5.49	0.61
Organic anion transporting polypeptide 3 (CHEMBL1743121)	OATP1B3	25	5.13	0.77
Multidrug resistance-associated protein 4 (CHEMBL1743128)	MRP4	106	4.70	0.47
Multidrug resistance-associated protein 2 (CHEMBL5748)	MRP2	57	4.69	0.42
Bile salt export pump (CHEMBL6020)	BSEP	361	4.68	0.51
Multidrug resistance-associated protein 3 (CHEMBL5918)	MRP3	43	4.52	0.45

a *N* is the number of compounds in the training series of each LLM.

**Figure 1:**
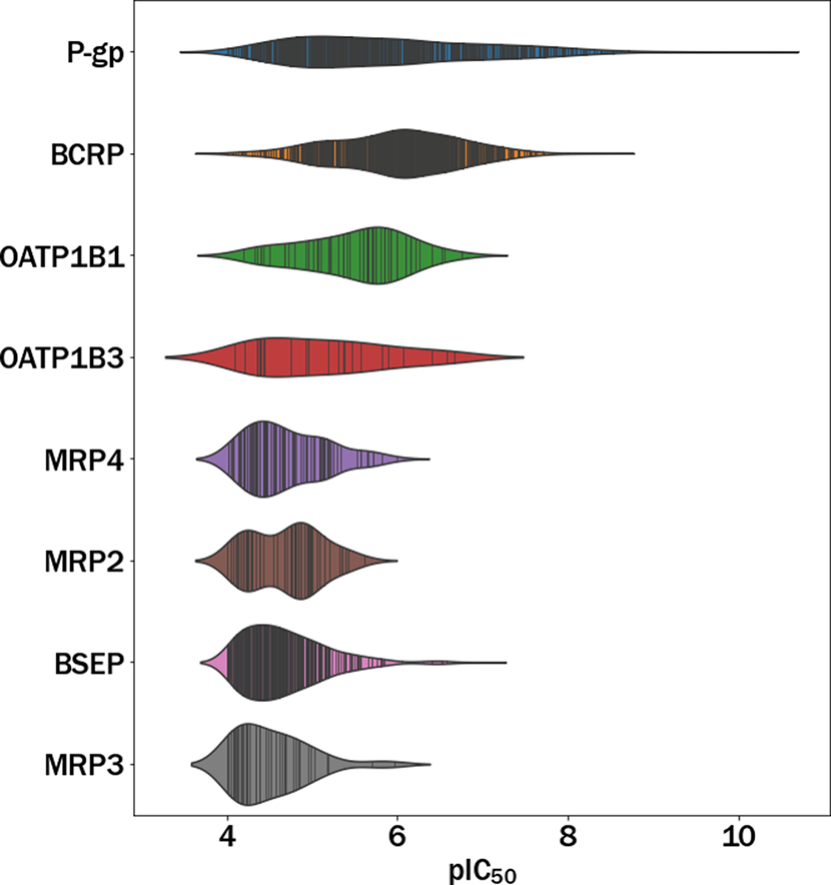
Violin plots of the pIC_50_ distributions of the eight hepatic selected transporters.

Figure 1 displays violin plots showing the distributions of pIC_50_ values for the selected transporters. Consistent with the information in Table 1, the mean pIC_50_ falls within the range of 4.5–6 for each transporter, with P-gp and BCRP exhibiting the highest means. Likewise, P-gp also exhibited the highest standard deviation, likely due to the inclusion of a larger number of diverse assays conducted on this particular transporter.

For each LLM, we obtained all compounds with IC_50_ annotations and developed a QSAR model using the pIC_50_ as the dependent variable. Morgan fingerprints (FPs) (nbits = 2048, radius = 2, features = enabled) were computed using RDKit 2019.9.3 (Landrum 2016) and used as input variables for building four machine learning (ML) regression models for each LLM with scikit-learn version 0.24.1;^39^ XGBoost 1.4.2 (XGB),^40^ Random Forest (RF),^41^ K-nearest neighbors (KNN),^42^ and Support Vector Machines (SVM).^43^ All models were trained using a grid search with 5-fold cross-validation (CV) to find the best hyperparameters based on the Mean Absolute Error (MAE) as the scoring metric. The model with the lowest MAE was selected for each of the LLMs. As part of our proposed hyperparameter grid for the SVM model, we included the linear kernel as an option in addition to the radial kernel, serving as an alternative to linear models. To ensure the robustness of the models, a 20-Repeated 5-fold CV approach was employed for the model evaluation. The selection of 20 repetitions was made considering that a Repeated k-fold CV requires fewer replicates than the total number of compounds available. As there were only 25 compounds collected for inhibiting the OATP1B3 transporter, 20 replicates were selected for the analysis throughout the entire article to maintain consistency in the methodology when using Repeated k-fold CV.^44^ The information corresponding to the settings of these models is provided in Supporting Information Table S1.

These models (LLMs) were used to predict eight transporter pIC_50_ values for the 437 compounds belonging to the cholestasis data set. For compounds with known experimental activity, the mean of all available experimental values was used instead of the predictions. The final matrix contains 437 rows (compounds) and 8 columns (transporters).

### *In Vitro* to *In Vivo* Extrapolations

*In vivo* half-maximal inhibitory equivalent oral doses (IEOD_50_’s) were calculated from the IC_50_ values by applying QIVIVE methods, translating concentrations into *in vivo* doses. For calculating IEOD_50_’s, we used the High-Throughput Toxicokinetics (httk 2.1.0) library.^45^ Monocompartmental (MC) models were built by using the default parameters provided by the httk library. Order 1 kinetics assumes that the drug concentration in the body can be described by a single compartment, which is appropriate for drugs that distribute rapidly and evenly throughout the body, under the assumption that the effect of a peripheral distribution is negligible at a steady state. QSAR models usually assume that the compound is at a steady state without considering any time-dependent processes that may affect the drug concentration. Hence, after computing the steady-state concentrations (C_ss_’s) using the MC model, the next step was to calculate the IEOD_50_ for each drug that inhibits each transporter. The IEOD_50_ is directly proportional to the *in vitro* IC_50_ and inversely proportional to the C_ss._^46^

The httk library^45^ can compute the percentile of the specified IEOD_50_ for the model. In our case, we obtained the 90th percentile, as the larger the percentile predicted C_ss_ from the MC model, the lower the IEOD_50_, due to the inverse relationship between C_ss_ and IEOD_50_. This approach is considered to be the most conservative, as cholestasis is a dose-dependent adverse outcome, and any compound with a therapeutic dose (T_D_) above the highest IEOD_50_ among the selected transporters would be considered cholestatic. The information about the therapeutic doses was obtained by matching the Drugbank IDs in the cholestasis data set with the corresponding entries in the Drugbank database (other sources of information consulted were drugs [https://www.drugs.com/] and Medscape [https://reference.medscape.com/]).

**Figure 2:**
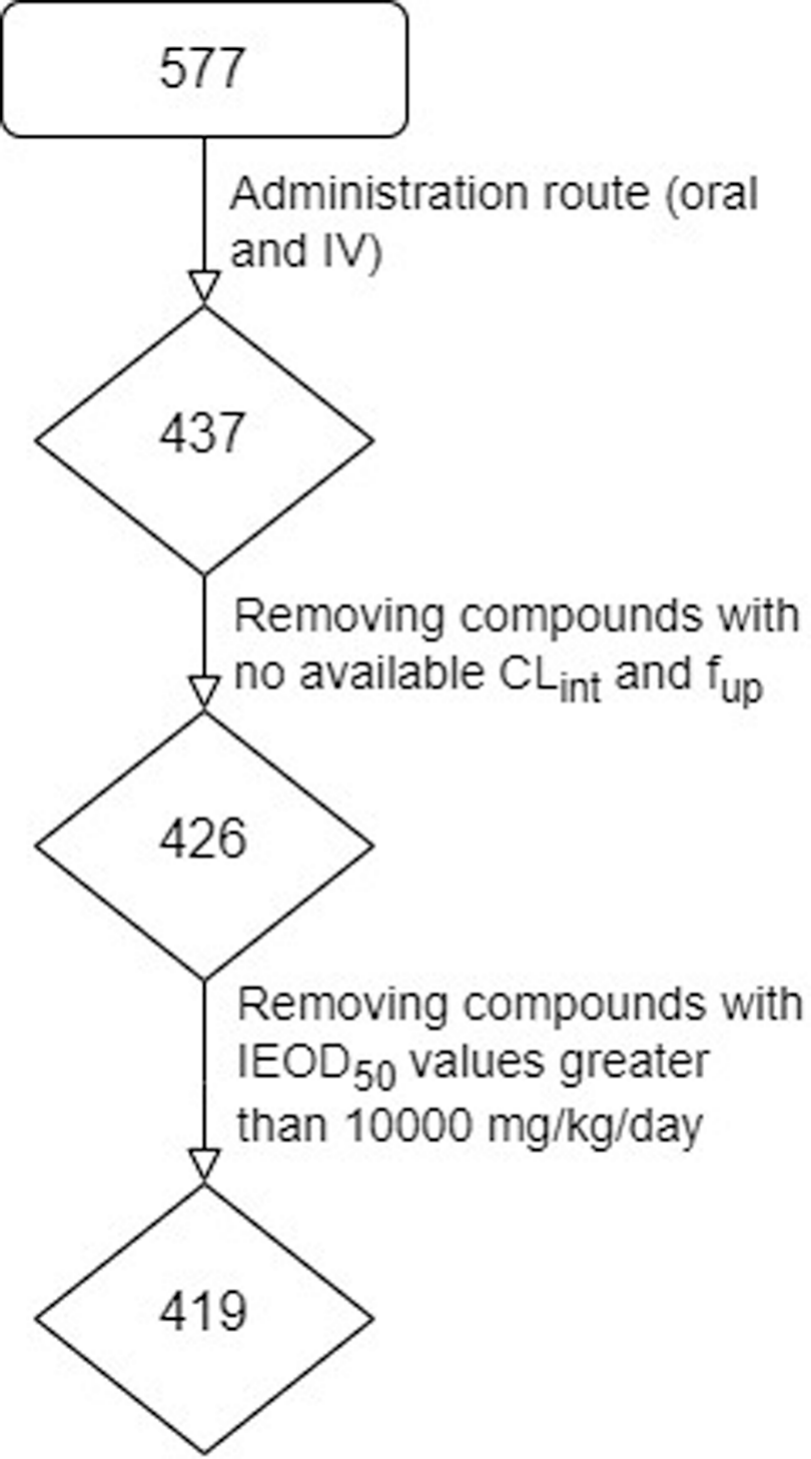
Cholestasis data set filters.

The calculation of IEOD_50_ requires obtaining physicochemical parameters such as molecular weight (MolWt), log P (octanol–water partition coefficient), and PK parameters such as intrinsic clearance (Cl_int_) and plasma-unbound fraction (f_ub_). For the compounds in the cholestasis data set, MolWt and log P were computed using RDKit. Experimental Cl_int_ and f_ub_ values were extracted from the httk databases (only drugs that have been experimentally tested with human hepatocyte cells), using DTXSIDs to identify the compounds in both data sets whenever possible. For the rest of the compounds, these values were predicted using OPERA version 2.9.^47^ Compounds that were unable to have either f_ub_ or Cl_int_ values calculated by OPERA were eliminated from the cholestasis data set. As a result, the data set contained a total of 426 compounds, with 115 classified as positive and 311 as negative. Finally, compounds with IEOD_50_ values larger than 10000 mg/kg/day (only 7 compounds were in this category) were removed from the cholestasis data set since we consider that these doses are not realistic from a physiological point of view. After this elimination, the final data set was reduced to 419 compounds (114 positives and 305 negatives). To further clarify the procedure, the filtering steps are summarized in Figure 2.

### Cholestasis Model Building

The cholestasis models were built using the series described above, using two different strategies: direct QSAR modeling and combining the predictions provided by the LLM (obtaining a metamodel). In the latter approach, we generated two different metamodels for assessing the advantages of incorporating PK information. In the metamodel incorporating PK information (Metamodel_pk), a compound was considered positive when its T_D_ was *n* times higher than the predicted IEOD_50_ for any of the considered transporter, where *n* is a factor adjusted to balance the sensitivity and specificity of the metamodel. Regarding the metamodel not incorporating PK information (Metamodel_not_pk), this model used IC_50_ information exclusively. A compound was classified as positive if the IC_50_ of any of the transporters was ≤300 μM, according to refs (48−51). Both metamodels were constructed as scikit-learn estimators to fully utilize the functionalities of the scikit-learn library.

Direct QSAR models were built using physicochemical descriptors (PC) and FP as predictor variables and were obtained using RDKit. The following algorithms were used: XGB, RF, Naïve Bayes approach (Multinomial Naïve Bayes [MNB] for FP and Gaussian Naïve Bayes [GNB] for PC descriptors),^52^ and SVM. Like in the regression models, we added the linear kernel within the hyperparameter grid for SVM classification models.

In order to find the best hyperparameters, all models underwent a grid search with a 5-fold CV, utilizing the Area Under the Receiver Operating Characteristic Curve (ROC AUC) score as the scoring metric. For the QSAR models, the algorithm achieving the highest ROC AUC among the four tested models was chosen as the optimal choice (Tables S2 and S3 provide further details).

### Model Evaluation

The model quality was evaluated using the model’s sensitivity (S), specificity (SP), accuracy (A), Matthews correlation coefficient (MCC), and ROC AUC.

#### Comparison between Repeated k-fold and “Similarity-Based Cross-validation” Performances

To incorporate the similarity in the assessment of the model predictivity, we compared the results obtained with a standard 20-Repeated 5-fold CV^44^ with a modified CV algorithm where the groups contain structurally dissimilar compounds. So, if the predictive power of a model is lower when using the “similarity-based CV”, this would indicate that it is worse for predicting when the compounds in the test series are more structurally different from those in the training series. For this modified version of CV, we applied hierarchical clustering to obtain five clusters (Cluster 1 = 51 compounds, Cluster 2 = 174 compounds, Cluster 3 = 61 compounds, Cluster 4 = 79 compounds, and Cluster 5 = 54 compounds) using fingerprints as input variables and the Jaccard distance as the evaluation metric. The same number of folds was established for both types of CVs to allow for a fair comparison. Each fold in the similarity 5-fold CV was trained using four clusters and validated with the remaining cluster, thus predicting compounds with low structural similarity to the training set of that fold. For detailed information on the search for optimal hyperparameter sets using both 20-Repeated 5-fold CV (Table S2.A in the Supporting Information) and similarity 5-fold CV (Table S2.B in the Supporting Information), refer to the Supporting Information. These tables provide further insights into the process of identifying the best hyperparameters for both types of cross-validation.

Likewise, intra- and intercluster similarities were evaluated using FP descriptors and the Tanimoto similarity metric. The mean similarity value of the three most similar compounds was computed intra- and intercluster. The Supporting Information’s Figure S1 presents a heatmap displaying the Tanimoto similarity values for both intra- and intercluster comparisons. The intracluster similarity values showed minimal differences, ranging from 0.41 (Cluster 2) to 0.47 (Cluster 1). Regarding the intercluster comparison, the similarity values ranged from 0.17 (Cluster 1–Cluster 5) to 0.31 (Cluster 2–Cluster 4). The observed results suggest that intracluster similarity outweighed intercluster similarity, indicating that this methodology has the potential to be worthy in evaluating the structural independence of the proposed approach.

#### Performances According to the “ATC-Based Cross-validation”

As mentioned above, our study intends to determine if the proposed methodology has advantages with respect to other approaches in terms of predictive quality when the compounds are different from those in the models’ training series. With this aim, complementing the “similarity-based cross-validation” described above, we applied a cross-validation procedure where drugs used in certain therapeutic areas (as identified by their ATC codes) are used to predict compounds used in different therapeutic areas. We started by compiling the ATCs for the compounds in our series for the five most represented ATCs: J01 (antibacterials for systemic use), N05 (psycholeptics), L01 (antineoplastic agents), C01 (cardiac therapy), and N02 (analgesics), as shown in Table 2. So, we conducted an ATC 5-fold CV, where each fold involved training on compounds from four of the five ATCs and predicting the validation set of compounds from the remaining ATC. Additional information regarding the optimal hyperparameters for each evaluated model can be found in Table S3 of the Supporting Information. Also, we calculated the intra- and inter-ATC group similarities in the same way as before used for computing the similarities described above for the similarity 5-fold CV method. Within the same ATC code, molecular similarities ranged from 0.22 (L01) to 0.54 (J01), as shown in Figure S2 of the Supporting Information. When comparing compounds from different ATC codes, similarities ranged from 0.16 (J01-C01, N05-J01, and L01-C01) to 0.27 (N05-N02). Similarly, to our previous CV strategy, intra-ATC similarity was found to be higher than inter-ATC similarity, justifying the use of this evaluation method, following the same approach as the previous one, for building models based on splits with high dissimilarity. This allows us to further verify that the performance of our proposed model is less dependent on the structural similarity between the training and test series than a direct QSAR model.

**Table 2:** Summary Information on the Top Five ATC Codes

**ATC**	**Number of compounds by class**	**Most common pharmacological groups**
J01 (antibacterials for systemic use)	# Cholestatic compounds = 20	β-lactams and penicillins
	# Noncholestatic compounds = 17	
N05 (psycholeptics)	# Cholestatic compounds = 12	Psycholeptics and hypnotics
	# Noncholestatic compounds = 25	
L01 (antineoplastic agents)	# Cholestatic compounds = 6	Alkylating agents ad plant alkaloids
	# Noncholestatic compounds = 16	
C01 (cardiac therapy)	# Cholestatic compounds = 3	Cardiac stimulants and antiarrhymics
	# Noncholestatic compounds = 15	
N02 (analgesics)	# Cholestatic compounds = 2	Antimigraine and opioids
	# Noncholestatic compounds = 15	

### Statistical Analyses

Student’s *t* tests at a 95% confidence level were used to determine whether there are statistically significant differences in the “Lipinski’s rules of five”^58^ variables between the positive and negative classes. This analysis was complemented with a two-way ANOVA to determine whether the effect of the transported type and the target class (as fixed factors) have a statistically significant effect on the IEOD_50_ at a 95% confidence level.

### Software

Table 3 shows a summary of the main software libraries and packages used in this study.

**Table 3:** Packages with Their Version Used and Main Applicability

**Package**	**Version**	**Applicability**	**Language**	**References**
scikit-learn	0.24.1	ML	python 3.6.13	(39)
numpy	1.19.5	Vector operations		(53)
statsmodels	0.12.2	Statistics		(54)
seaborn	0.11.1	Visualization		(55)
matplotlib	3.3.4	Visualization		(56)
RDKit	2019.9.3	Chemical		(38)
pandas	1.1.5	Dataframe operations		(57)
chembl-webresource-client	0.10.8	ChEMBL requests		(32)
PubChemPy	1.0.4	PubChem requests		(33)
MedCode	1.3	ATC codes		(36)
XGBoost	1.4.2	Boosting model		(40)
httk	2.1.0	Pharmacokinetic	R 4.2.1	(45)

## Results and Discussion

### Overview

To detect potential differences between cholestatic and not cholestatic compounds due to physicochemical properties, we run a preliminary study using some of the variables used by “Lipinski’s rules of five”.^58^

**Figure 3:**
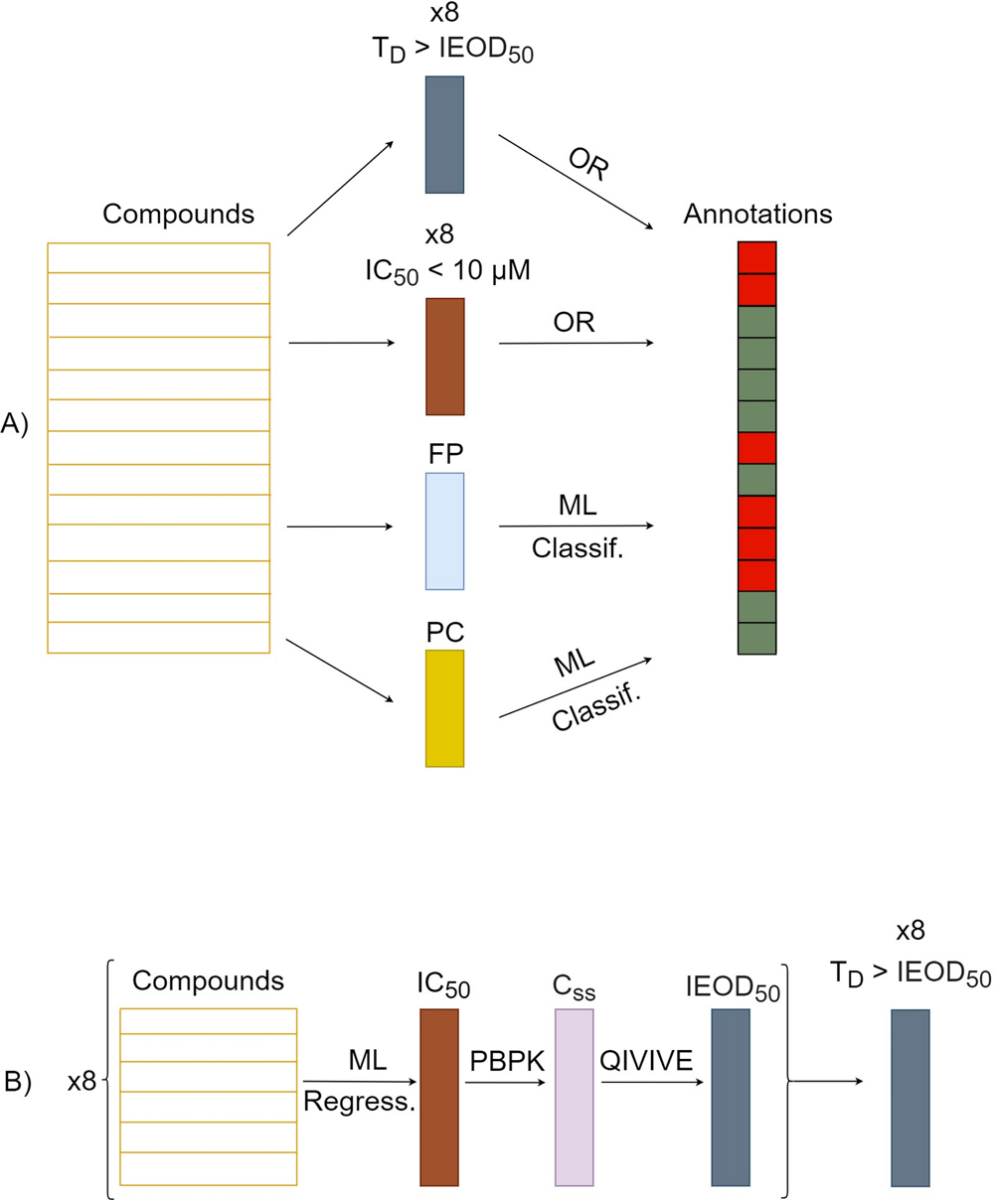
Scheme of the proposed methodology. (A) High-level view of the four models being compared: Metamodel with PK information (gray), Metamodel without PK information (brown), direct QSAR with FP descriptors (light blue), and direct QSAR with PC (yellow). Red cells represent cholestatic compounds, and green cells represent noncholestatic compounds. (B) Scheme of the proposed workflow to introduce toxicokinetics in the modeling.

The approach described here was based on physiological knowledge, where we constructed models for simpler phenomena (MIEs) that represent relevant components of the complex end point (AO) and combined the predictions incorporating toxicokinetic considerations. We started by gathering existing information on the biological processes involved in this end point from the AOP wiki. Then, we developed QSAR models for each of the hepatic transporters identified as relevant MIEs: P-gp, BCRP, OATP1B1, OATP1B3, MRP4, MRP2, BSEP, and MRP3 (see Table 1), as described in the Materials and Methods section. The predicted *in vitro* inhibitory information was exploited by applying logical rules. A simple logical OR on this prediction matrix was used to label compounds showing inhibitory activity for any of these transporters as potential cholestatic compounds (second part of Figure 3.A).

However, this approach has the limitation that the inhibitory *in vitro* concentrations obtained by the models can be nonrepresentative of the ones reached in therapeutics due to differences in clearance, protein binding, bioavailability, and other pharmacokinetic parameters. For this reason, we incorporated toxicokinetic considerations to obtain IEOD_50_’s (representing *in vivo* doses) from the predicted IC_50_’s (representing *in vitro* data) using QIVIVE models. The proposed workflow (Figure 3.B) starts with applying a PBPK model to obtain C_ss_ from the input pIC_50_. Then, the QIVIVE approach allows obtaining IEOD_50_ from the C_ss_. Finally, the IEOD_50_’s are compared with T_D_ (obtained from public sources, as described in the Materials and Methods section), and we used a logical OR rule to label as cholestatic the compounds for which the T_D_ is larger than the highest IEOD_50_ among all transporters (first part of Figure 3.A).

The results obtained using this approach were compared with a classical direct modeling method that uses compound structures to build QSAR models, using both FP and PC descriptors (summarized in the third and fourth sections of Figure 3.A), and predicted cholestasis directly without considering any mechanistic information.

The comparison of our approach with the direct QSAR models included not only an analysis of their performance using standard metrics but also an additional analysis for comparing their applicability for the prediction of dissimilar compounds. This involves an evaluation of their predictive quality using “similarity-based” and “ATC-based” complementary CVs, carried out as described in the Materials and Methods section.

### Preliminary Analyses

**Figure 4:**
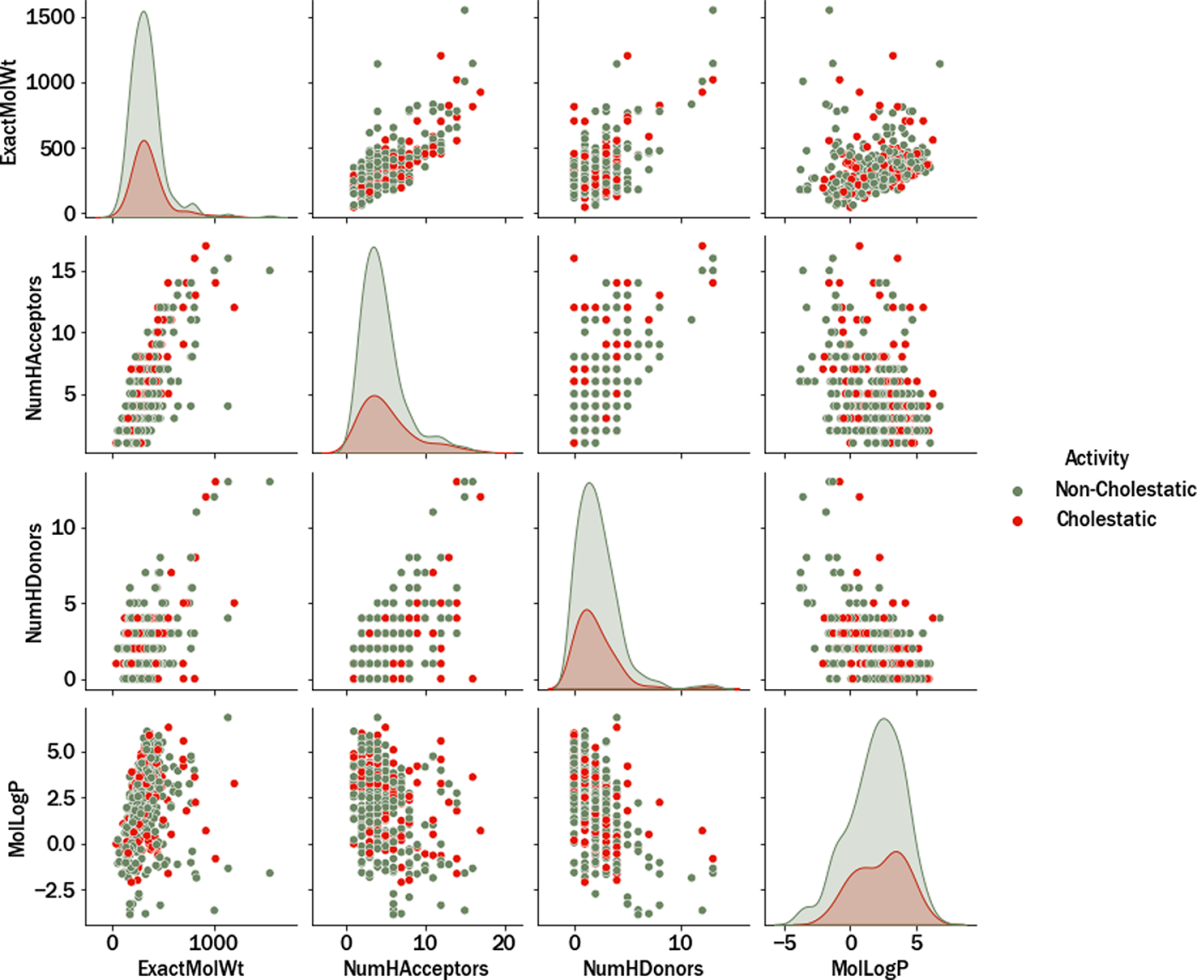
Distribution of Lipinski’s rules of five: Molecular weight (ExactMolWt), Number of hydrogen bond donors (NumHDonors), Number of hydrogen bond acceptors (NumHAcceptors), and octanol–water partition coefficient log P (MolLogP).

As a preliminary step, we studied possible differences in the physicochemical properties between the cholestatic and noncholestatic compounds in the studied series of 419 compounds. Figure 4 shows the summary of density and scatter plots, separated by class for the compound’s molecular weight (ExactMolWt), number of hydrogen bond acceptors (NumHAcceptors), number of hydrogen bond donors (NumHDonors), and log P (MolLogP). These are the properties represented by Lipinski’s rules of five,^58^ which are known to describe important properties for the pharmacokinetic and pharmacodynamic characteristics of compounds. The center of the distribution is slightly higher for ExactMolWt, MolLogP, and NumHAcceptors in the set of cholestatic molecules compared to the noncholestatic ones. However, according to the Student’s *t* test performed, the differences were not statistically significant for any of the properties studied at a 95% confidence level.

#### Low-Level Models

**Figure 5:**
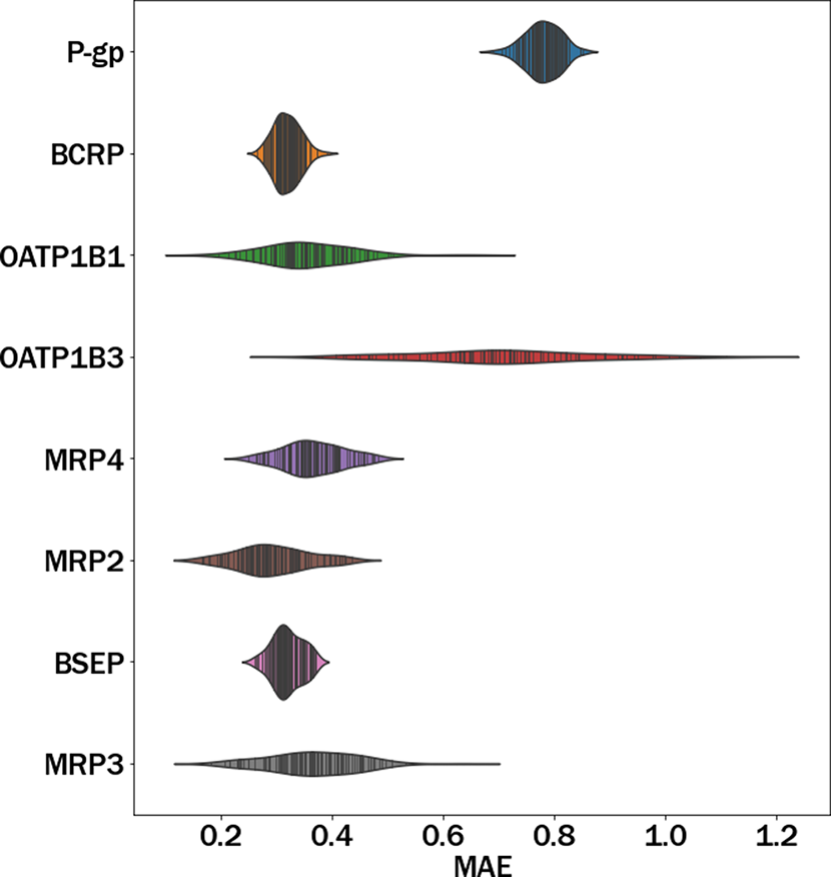
Violin plot with the MAE obtained for each LLM.

Individual QSAR regression models for the eight transporters selected (Table 1) were built as described in the Materials and Methods section. The violin plots in Figure 5 show the MAE distributions obtained from the 20-Repeated 5-CV for each of the eight low-level models. Particularly, the models for P-gp and OATP1B3 inhibition had the poorest performance. Regarding deviations between folds, P-gp’s extensive data leads to minimal variations, while OATP1B3’s limited data results in significant deviations. These findings emphasize the impact of variability between several assays and data availability on predictive performance, such as described above.

Table 4 presents the mean and standard deviation of the 20 repetitions of the 5-fold CV. It reveals that P-gp (0.78) and OATP1B3 (0.68) had the highest mean MAEs, with a low standard deviation for P-gp (0.03) and a higher standard deviation for OATP1B3 (0.19). These observations align with the insights shared in Figure 5. The remaining transporters exhibited similar mean MAE values. The models for BCRP and BSEP demonstrated less deviation between folds (akin to what was observed for P-gp), as these models had more training data compared to the others.

**Table 4:** Mean and Standard Deviations of MAEs Obtained from the 20-Repeated 5-fold CV for the Eight Selected Transporters

**Metrics**	**BCRP**	**MRP2**	**MRP3**	**MRP4**	**OATP1B1**	**OATP1B3**	**BSEP**	**P-gp**
**MAE**_**mean**_	0.32	0.29	0.36	0.36	0.36	0.68	0.33	0.78
**MAE**_**std**_	0.02	0.06	0.08	0.05	0.07	0.19	0.03	0.03

**Figure 6:**
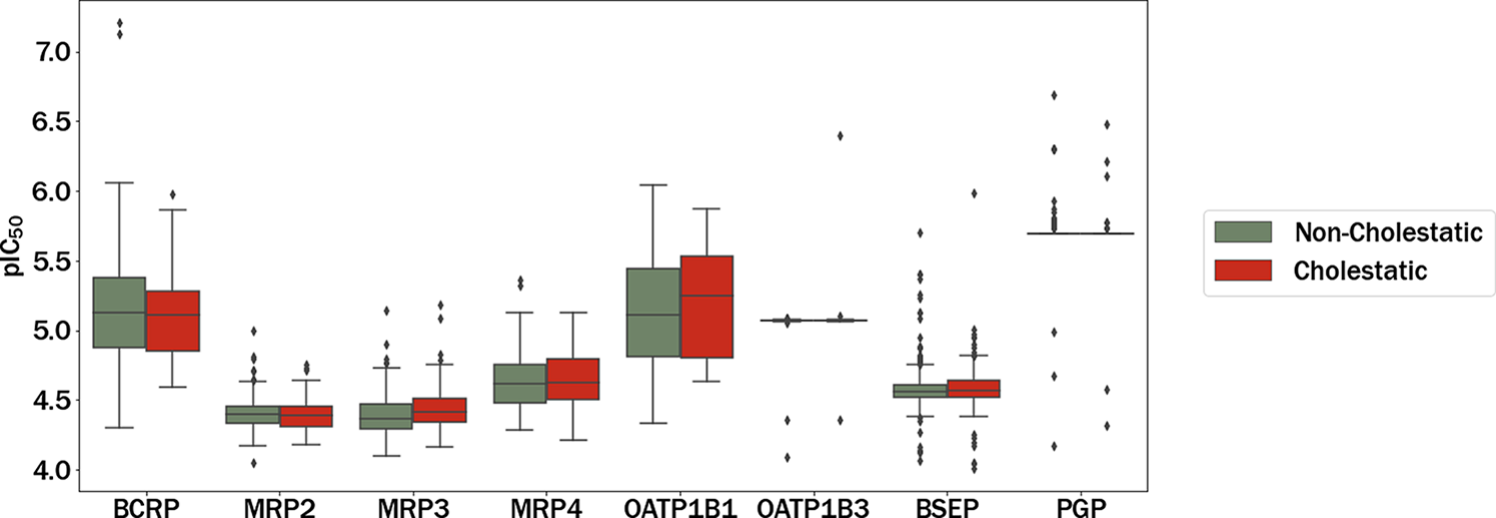
Box plots of the pIC_50_ distributions separated by class for each selected transporter.

Figure 6 depicts the box plots illustrating the predicted pIC_50_ distribution for each transporter. Notably, the worst performing models (P-gp and BCRP) exhibit similar values in their distributions. This finding could have the potential to impact the overall quality of the metamodels. It is important to note that these data impose an upper bound on the quality of the predictive models derived from them. While it could be tempting to push the model beyond this limit, doing so risks production of model overfitting, compromising their predictive performance. The analysis showed statistically significant differences (*p* < 0.01) in the pIC_50_ distributions between the different transporters and classes (two-way ANOVA, 95% confidence level, as described in the Materials and Methods section).

#### Incorporating TK Considerations

The predicted *in vitro* pIC_50_ cannot be expected to correlate directly with observed cholestatic outcomes without first transforming these to *in vivo* doses (IEOD_50_’s) and then comparing these doses with the ones administered in clinical use. The first step, the computation of IEOD_50_’s, was carried out using QIVIVE models, as described in the Materials and Methods section. To evaluate the predictive power of the models built by OPERA for predicting f_ub_ and Cl_int_, compounds of the cholestasis data set with experimental values (from queries to the httk library) were predicted. Figure S3 in the Supporting Information displays a scatter plot with the *X*-axis representing the experimental values extracted from httk and the *Y*-axis showing the OPERA predictions for the same compounds for both f_ub_ (Figure S3.A in the Supporting Information) and Cl_int_ (Figure S3.B in the Supporting Information). In this figure, minimal deviations between the actual values and the predictions can be observed. To further validate the predictive power, Table S4 in the Supporting Information presents the MAE values for both f_ub_ (MAE = 0.07) and Cl_int_ (MAE = 8.50), as well as the mean and standard deviation between the experimental values from httk and the predicted values from OPERA, which exhibit practically identical results. These results highlight the quality of the OPERA models in predicting pharmacokinetic parameters.

**Figure 7:**
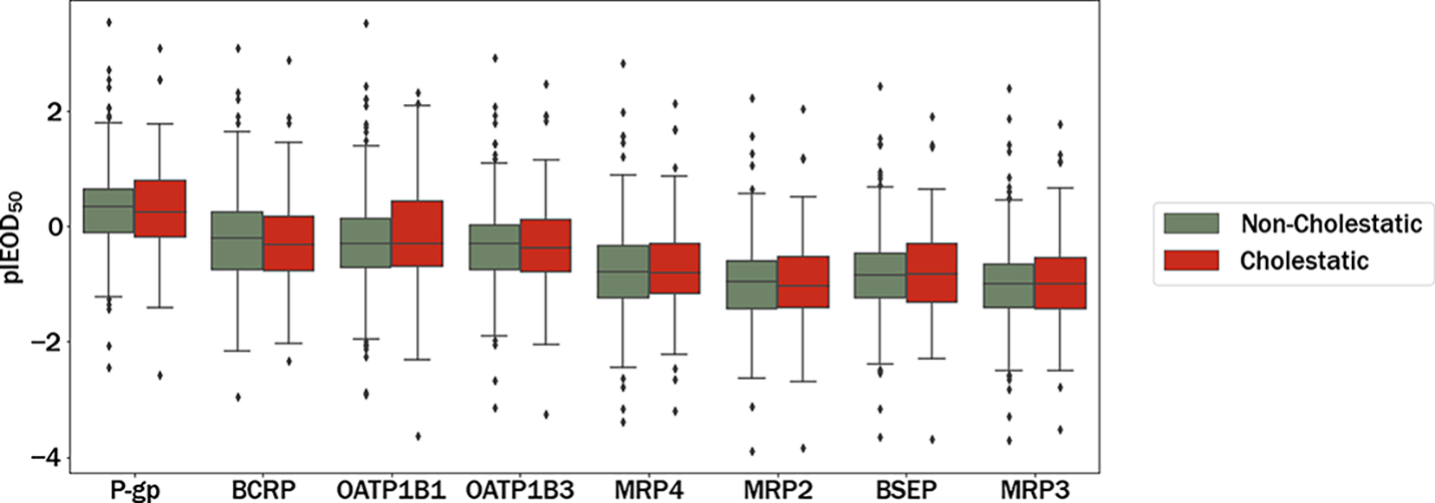
Box plots comparing the pIEOD_50_’s (potential of IEOD_50_) predicted for the series studied and the eight transporters separated by class. Transporters are shown in decreasing order with respect to the median pIEOD_50_.

Figure 7 shows box plots with the pIEOD_50_’s (−log_10_(IEOD_50_)) of the eight selected transporters for cholestatic and noncholestatic drugs, side by side. It can be seen that the median value of inhibitory potential for each transporter is nearly identical between cholestatic and noncholestatic compounds. The analysis showed no statistically significant differences (*p* < 0.05) in the pIEOD_50_ distributions between the different transporters and classes (two-way ANOVA, 95% confidence level, as described in the Materials and Methods section). The absence of statistical significance in the pIEOD_50_ distributions between different transporters and classes, in contrast to the statistical significance observed in the pIC_50_ distributions, may be due to the fact that *in vitro* models can oversimplify or fail to fully capture the complexity of metabolic pathways that occur *in vivo*. Therefore, based on *in vivo* extrapolations, the whole set of hepatic transporters could have the same relevance in predicting cholestasis.

### Predictive Quality of the Metamodels and the Direct QSAR Models

The metamodel obtained using PK information (Metamodel_pk) was constructed by using a logical OR to combine the presence of T_D_ higher than the IEOD_50_. In other words, a compound was predicted to be cholestatic if its T_D_ was higher than the predicted IEOD_50_ for any of the selected hepatic transporters. For the metamodel without PK information (Metamodel_not_pk), a compound was predicted as cholestatic if any transporters had an IC_50_ below 300 μM. The direct QSAR models were built using fingerprints and physicochemical descriptors as predictor variables. The models and approaches utilized in this article have undergone a meticulous grid search, aiming to identify the optimal set of hyperparameters, such as described in the Materials and Methods section.

#### Comparison between Repeated k-fold and “Similarity-Based Cross-validation” Performances

The results of a direct QSAR model coming from a Repeated k-fold may be too optimistic, and these results may not be representative of practical problems. One of the main reasons is that, in real drug development applications, the structure of the new drug candidate is often very different from the structures of the training series. Therefore, to obtain a fairer comparison, we further evaluated the predictive quality of our models by assessing if they could accurately predict the properties of structurally diverse compounds. With this aim, we applied a “similarity-based CV” (described in detail in the Materials and Methods section) where the series was split into five structurally dissimilar subgroups using hierarchical clustering. Then, we applied a similarity 5-fold CV where four subgroups were used to predict the remaining one, containing structurally dissimilar compounds. By comparing the predictive quality of this approach with those from a standard 20-Repeated 5-fold CV, where groups were assigned randomly, we can evaluate how dependent the structural similarity is on the prediction quality for all of the studied models.

Table 5 presents the mean and standard deviation for both the 20-Repeated 5-fold CV and the similarity 5-fold CV.

**Table 5:** Mean and Standard Deviations of the Sensitivity (S), Specificity (SP), AUC, MCC, and Accuracy (A) for Each Model in Both the 20-Repeated 5-fold CV and Similarity 5-fold CV

CV type	Model type	**S**	**S_std**	**SP**	**SP_std**	**AUC**	**AUC_std**	**MCC**	**MCC_std**	**A**	**A_std**
**20-Repeated 5-fold CV**	**Metamodel_pk**	0.84	0.07	0.55	0.06	0.69	0.05	0.34	0.19	0.63	0.05
	**Metamodel_not_pk**	1.00	0.00	0.00	0.01	0.50	0.00	0.01	0.03	0.27	0.04
	**QSAR_model_FP**	0.34	0.11	0.87	0.05	0.60	0.05	0.23	0.12	0.72	0.05
	**QSAR_model_PC**	0.32	0.10	0.84	0.06	0.58	0.06	0.11	0.12	0.69	0.05
**Similarity 5-fold CV**	**Metamodel_pk**	0.81	0.06	0.54	0.15	0.67	0.09	0.29	0.14	0.62	0.10
	**Metamodel_not_pk**	0.06	0.06	0.98	0.02	0.52	0.03	0.07	0.10	0.74	0.09
	**QSAR_model_FP**	0.20	0.09	0.94	0.03	0.57	0.04	0.19	0.10	0.75	0.09
	**QSAR_model_PC**	0.24	0.14	0.90	0.04	0.57	0.05	0.16	0.11	0.74	0.06

**Figure 8:**
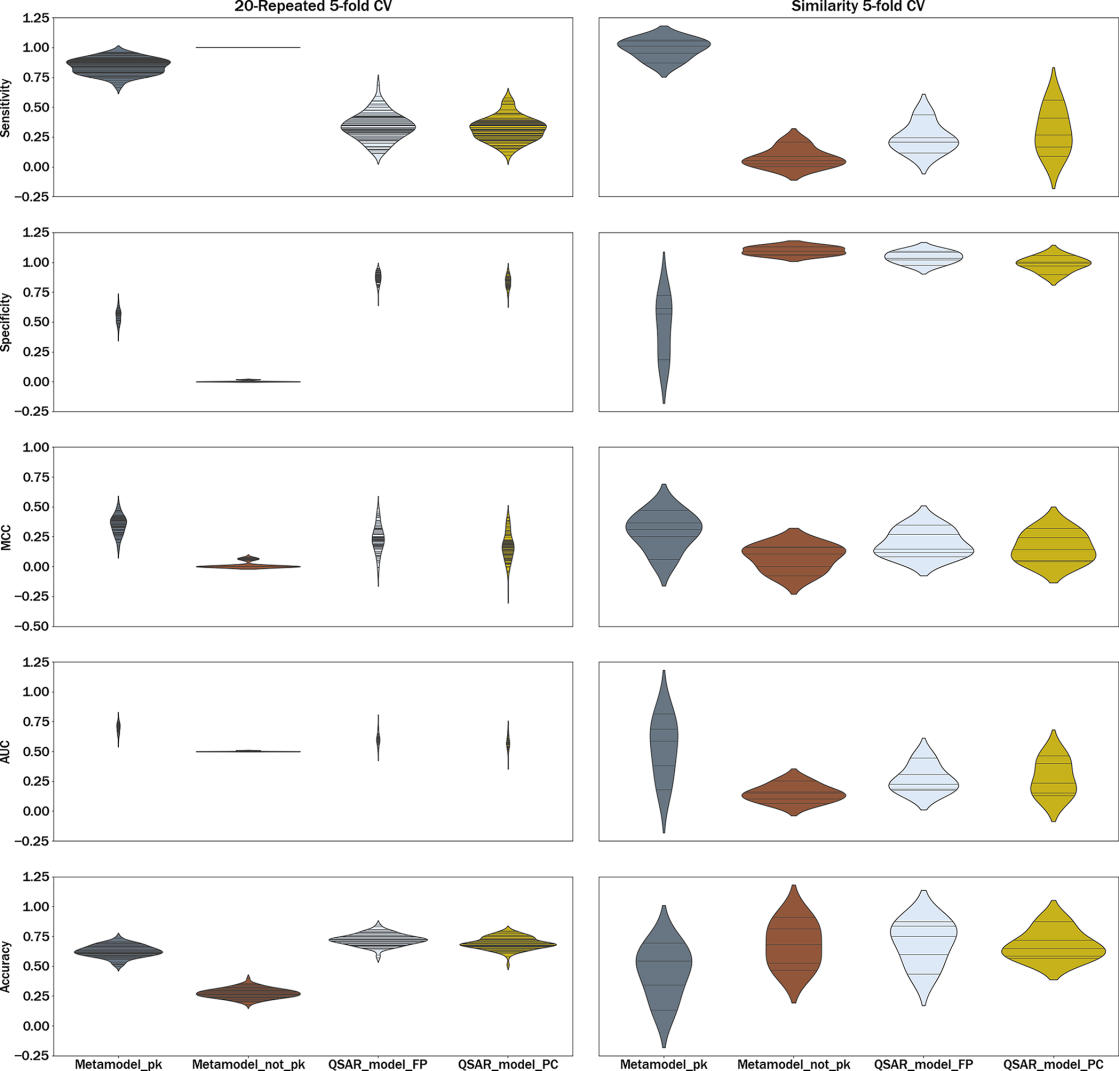
Sensitivity, specificity, MCC, AUC, and accuracy according to the 20-Repeated 5-fold CV (left column) and the Similarity 5-fold CV (right column).

Figure 8 displays violin plots for the sensitivity, specificity, MCC, AUC, and accuracy of each model based on the 20-Repeated 5-CV (left column) and the similarity 5-fold CV (right column). Additionally, Table 5 provides a summary of the mean and standard deviation of the metrics presented in Figure 8. This figure depicts how the average variability across folds was similar for both types of CV across different models (the std of the Metamodel_pk was lower than that of the QSAR models). The Metamodel_not_pk exhibited the least variability between folds for each metric.

The results observed in Figure 8 indicate that Metamodel_pk was not affected by the decrease in structural similarity, as its sensitivity remained above 0.80 in both scenarios (Table 5). Concerning specificity, Metamodel_pk did not exhibit any variations depending on the CV utilized, and its performance was the same regardless of the CV used (0.55 approximately). Here, it is important to clarify that the low performance in terms of the specificity of the metamodel with PK information could be due to the lower predictivity of LLMs with the worst performances (based on an analysis not included). For instance, the P-gp model only achieved correct predictions for the noncholestatic activity of compounds in 7% of the cases, and the OATP1B3 model achieved 15% of successes. These results may have an impact on the final quality of Metamodel_pk in terms of specificity. It should be noted that this observation is consistent with the description of the LLMs, where both transporter models exhibited the poorest performances.

In the first type of cross-validation (left column of Figure 8), Metamodel_not_pk achieved a sensitivity of 1.00. However, in the second type of cross-validation (right column of Figure 8), the sensitivity dropped to approximately 0.00. Interestingly, despite these variations in sensitivity, both types of cross-validation resulted in ROC AUC scores that were very close to 0.5. When comparing the two metamodels, it was observed that the model incorporating PK information exhibited significantly a higher ROC AUC score (between 0.15–0.19 higher for both CV approaches) compared to the model without PK information.

In the Repeated k-fold CV, the RF model showed the best performance for both QSAR models. However, when using Similarity k-fold CV, the XGB model outperformed the others in terms of sensitivity, MCC, and ROC AUC score. Thus, for QSAR models, we could say that the lower the structural similarity, the lower the sensitivity. Regarding the QSAR model utilizing FP descriptors, it consistently exhibited slightly higher specificity compared to the model using PC descriptors. Both models showed similar values of sensitivity, with 0.34 for QSAR_model_FP and 0.32 for QSAR_model_PC in the Repeated k-fold CV and 0.20 and 0.24, respectively, in the Similarity-based CV. Aggregated quality indexes such as the AUC or the MCC show an improvement in the overall predictive quality of Metamodel_pk. On the contrary, the accuracy is slightly better for QSAR_model_FP (about 0.7 for both kinds of CV), the most specific model, since the proportion of positive annotations is low (approximately one positive compound for every three negative compounds) and a specific model has fewer false positives. Considering the low proportion of positive compounds, more sensitive models (such as Metamodel_pk) are far more valuable and useful. Furthermore, Table S5 in the Supporting Information displays the evaluation of each metric for every model in each fold of the similarity 5-fold CV. In broad terms, regardless of the similarity space used, the metamodel incorporating PK information outperformed both QSAR models, showing a higher sensitivity, MCC, and AUC.

#### Performances According to the “ATC-Based Cross-validation.″

The analysis by ATC codes allows the categorization of drugs based on their therapeutic and pharmacological properties. In terms of structural independence, the ATC code can provide insight into the relationship between a drug’s structure and its therapeutic properties. In this study, the predictive quality of the models was further tested by using a CV approach that closely resembles the previous point but where the folds were obtained by grouping compounds with the same ATC code. This exercise aimed to check whether models trained with compounds from some therapeutic regions can accurately predict the toxicity of compounds belonging to different therapeutic areas, as characterized by their respective ATC codes.

**Figure 9:**
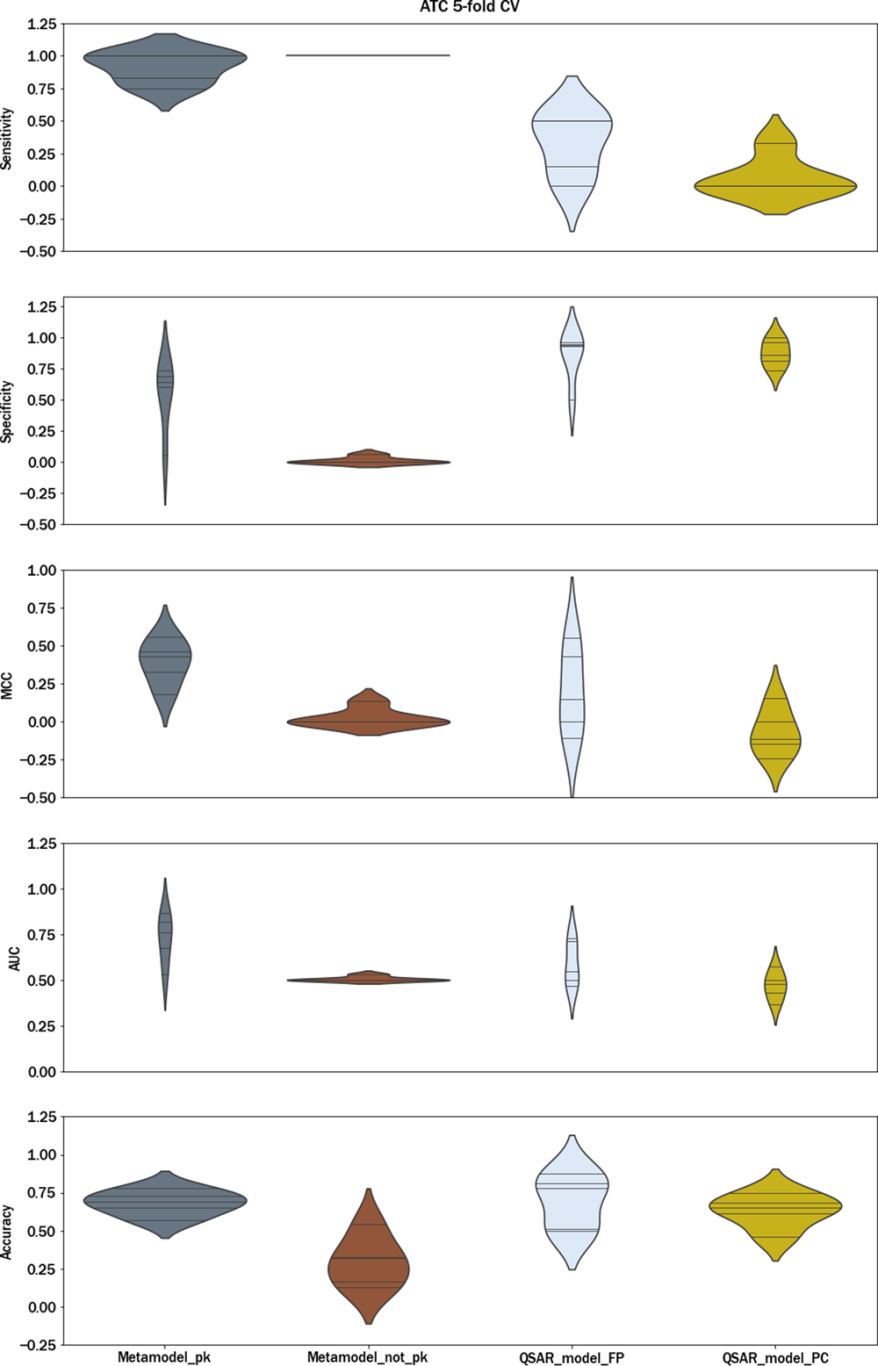
Sensitivity, specificity, MCC, AUC, and accuracy according to ATC 5-fold CV.

Figure 9 illustrates the performance of the different models, providing further insights into their comparative robustness to predict structurally diverse compounds thanks to the use of the ATC-based k-fold approach. Comprehensive details for each model and metric can be found in Table S6. Additionally, Table S7 presents a comprehensive breakdown of the results for each fold, enabling a granular examination of the model performance across multiple metrics. The results of Figure 9 show the same variability across folds for different models and metrics that was discussed in previous plots and the same trends with respect to the best models in terms of sensitivity and specificity. Regarding sensitivity, the best results were obtained for the similarity-based CV approach, with Metamodel_pk exhibiting much higher sensitivity (0.92) than other models. Overall, the metamodel outperformed other models in all evaluated metrics except for specificity and accuracy, as previously mentioned in the section on Similarity-based CV. Analyzing each fold based on Table S7 in the Supporting Information and comparing the MCC scores of different models, we observe that Metamodel_pk achieved notably good MCC values (specifically for the ATC code of anticancer drugs (MCC_L01_ = 0.47) and cardiovascular therapy (MCC_C01_ = 0.56), whereas the QSAR models displayed MCC scores ranging from −0.24 to 0.15, depending on the selected QSAR model. Indeed, these two ATC groups (L01 and C01) frequently exhibit diverse molecular scaffolds, posing a greater challenge when attempting to predict them using models trained with other ATC groups. The inherent dissimilarity between these groups adds an additional layer of complexity to the prediction task. All of this highlights the big drawback of QSAR models and provides evidence that our methodology can address this issue by bridging the gap created by conventional models.

## Discussion

The methodology presented here allows the prediction of cholestasis using an alternative approach to the direct QSAR models, which integrates mechanistic information and pharmacokinetic properties. To effectively execute this methodology, it is necessary to build low-level models that predict the IC_50_ of each inhibited transporter with the utmost precision. These *in vitro* concentrations are subsequently extrapolated to IEOD_50_’s through the QIVIVE models. Similarly, accurate models are essential for determining the experimental and physicochemical parameters that are used to feed the monocompartmental model underlying the calculation of C_ss_ in the QIVIVE models.

Determining whether one model’s predictive power surpasses another depends on the intended uses of the prediction results. Achieving an optimal balance between sensitivity and specificity is crucial in some scenarios. However, in discovery and early drug development, the main goal of *in silico* studies is the early detection of the potentially toxic compound. Therefore, a lower specificity is much preferable to a lower sensitivity. This is particularly true in the case of cholestasis since, as previously mentioned, it is a severely adverse event and a relevant mechanism of DILI, which is one of the primary causes of drug withdrawal or termination of clinical trials. Hence, in new drug development, sacrificing potential candidates may be preferable to avoid future financial losses of millions of dollars.

It is worth noting that the IC_50_ values for many compounds were predicted, as their experimental values were unknown. The LLMs exhibited similar performances, except for the P-gp and OATP1B3 transporters. In the case of the P-gp model, its significant assay variability, as described by other authors,^36,59^ directly impacts the measurement of compound biological activity, resulting in the lowest-performing low-level model. Similarly, the limited number of compounds available to train the OATP1B3 model contributes to its consistently poor performance across different splits of the 20-Repeated 5-fold CV. With regard to the P-gp model, we could have applied stricter criteria to the assays to include more homogeneous data. However, to maintain methodological consistency, the same procedure would need to be applied to other transporters, potentially resulting in an extremely small number of compounds. Regarding the OATP1B3 transporter, for which it was not possible to build a good model, it could have been excluded from the metamodel. However, we preferred to keep it to maintain a more complete representation of all the targets involved in the biological mechanisms underlying cholestasis occurrence. Therefore, we opted for a controlled risk approach, monitoring subsequent evaluations where metamodel failures were observed. Therefore, the inaccuracy of the predicted values should be borne in mind, as it limits the quality of the model. Even so, we consider that even a rough estimation of the pIC_50_ is likely to improve the overall predictive performance of the method, and it has value in exemplifying an approach which can be much improved when higher-quality estimations of these parameters (either experimental or predicted) can be generated.

In evaluating the predictive power of the selected strategies (Figures 8 and 9), it was found that Metamodel_pk exhibited substantially greater sensitivity than Metamodel_not_pk. The improved sensitivity is likely due to PK data providing information about a drug’s behavior in the body, including ADME processes and drug’s exposure. The model that did not incorporate PK (Metamodel_not_pk) information only used *in vitro* data, which cannot accurately predict how a drug will behave *in vivo*. Similar findings were observed when comparing Metamodel_pk to either of the two classical QSAR models, with the first model demonstrating higher sensitivity than the QSAR models.

The predictive performance of QSAR models and metamodels was further investigated by evaluating their quality in situations where the validation sets have a lower resemblance to the training sets (Figure 8). Our results confirmed the theory that QSAR models are highly dependent on the structural similarity between the test series compounds and the ones in the training series. This highlights the need for careful consideration of the selection of the compounds used in the training set and the evaluation of the performance of the QSAR models for structurally diverse compounds. In contrast, our results showed that the metamodel based on PK information was not dependent on structural similarity, probably because it better represents the underlying biological mechanisms. Overall, our study provides further evidence for the differential performance of QSAR models and metamodels in predicting cholestasis and highlights the importance of considering the structural similarity of the validation compounds when evaluating the predictive quality of QSAR models. These findings suggest that the metamodel with PK information is much less dependent than the direct QSAR models of the structural similarity and, therefore, can produce a better prediction for original structures, which is one of the common use scenarios in drug discovery.

This hypothesis was further confirmed using an additional validation analysis, in which we predicted compounds of a certain therapeutic area (as characterized by their ATC codes) using compounds from other therapeutic areas. In this type of analysis, we are aware that alternative strategies, such as clustering scaffolds, could be employed. However, this approach may introduce the challenge of having an abundance of scaffolds that may not be relevant to the analysis, requiring manual selection. Thus, by selecting the five most prevalent ATC groups within the data set under investigation, we ensure a more objective analysis. The analysis revealed that the metamodel with PK information exhibited significantly higher sensitivity across all ATC groups than did the QSAR model. Concerning the groups J01, N05, and N02, the MCC was not much superior for the metamodel with respect to QSAR_model_FP (Table S7 of the Supporting Information). However, the metamodel with PK information demonstrated clear benefits in terms of predictive quality for the following L01 and C01 ATC subgroups. This could be due to the fact that compounds belonging to ATC groups, such as antineoplastic or cardiac therapy drugs, do not usually share common scaffolds with other ATC groups. Once more, this emphasizes the recommended methodology for cases where the objective is to predict the cholestasis activity of a novel drug from a therapeutic category with many structural differences.

The results indicate that the metamodel incorporating PK information is better for typical applications in discovery or early development stages’ toxicity assessment than the direct models and Metamodel_not_pk. This could be explained by the fact that the metamodel with PK information considers both hazard and exposure, providing a more comprehensive representation of the underlying biological mechanisms of action.

## Conclusion

Here, we present an innovative methodology integrating multiple biological phenomena (MIE) with pharmacokinetic properties (QIVIVE) to predict cholestasis as a more comprehensive approach to the phenomena of interest. The aim was to assess the predictive ability of the proposed methodology and direct QSAR modeling in three different ways: by evaluating the overall performance of the models (S, SP, MCC, ROC AUC, A) using classical methods as well as by using ad-hoc cross-validation approaches where the predicted compounds are selected to have low similarity (in terms of structural similarity or ATC codes) with the training series.

After comparing the predictive power of the proposed models, it was determined that, in broad terms, the metamodel with PK information outperformed both the metamodel without PK information and QSAR models in terms of sensitivity, MCC, and ROC AUC. Nevertheless, concerning accuracy, the outcomes were less favorable than those of the QSAR models. This outcome is understandable since there were significantly more negative compounds than positive ones, and ML models often predict the majority class more accurately due to higher information available, resulting in a slightly lower hit rate.

The Metamodel_pk showed structural independence, as its sensitivity and specificity remained unchanged regardless of the similarity space tested (using the “similarity-based CV”). In contrast, the QSAR models showed decreasing sensitivity as similarity decreased. In the “ATC-based CV”, Metamodel_pk also showed a much higher sensitivity than the QSAR model, especially in cases where there are more diverse structures that maintain a lower structural similarity, as in the case of antineoplastic agents (L01) or cardiac therapy (C01). Overall, the metamodel that included PK information demonstrated superior predictive performance for more diverse structures and cholestatic compounds.

In light of these results, we propose that this methodology can be applied to other complex toxicological end points, aiding experts in developing new frameworks to support NGRA as it considers both hazard and exposure for a more comprehensive toxicity assessment.

## Data Availability

The code and data sets used in the study are publicly available from the GitHub repository: https://github.com/phi-grib/Cholestasis_paper.

## Supporting Information Available

Low-level models optimization; 20 repeated 5-fold CV, ATC 5-fold CV and similarity 5-CV models optimization; Tanimoto similarity values for intra- and interclusters and intra- and inter-ATC; f_ub_ and CL_int_ performances; similarity 5-fold CV and ATC 5-fold CV performances by fold; and ATC 5-fold CV mean performances. (PDF)
