# Supplementary material for: Integrating Mechanistic and Toxicokinetic Information in Predictive Models of Cholestasis
Source: J Chem Inf Model. 2023 Sep 3;64(7):2775–88. doi: 10.1021/acs.jcim.3c00945 (PMC11005038; doi:10.1021/acs.jcim.3c00945)
Supplement: Supplementary file 1 — ci3c00945_si_001.pdf [file ci3c00945_si_001.pdf]

Supporting information

Integrating mechanistic and toxicokinetic information in predictive models of cholestasis

Pablo Rodríguez-Belenguer[1,2], Victor Mangas-Sanjuan[2,3], Emilio Soria-Olivas[4], Manuel Pastor[1]

[1]Research Programme on Biomedical Informatics (GRIB), Department of Medicine and Life Sciences, Universitat Pompeu Fabra, Hospital del Mar Medical Research Institute, 08003 Barcelona, Spain

[2]Department of Pharmacy and Pharmaceutical Technology and Parasitology, Universitat de València, 46100 Valencia, Spain

[3]Interuniversity Research Institute for Molecular Recognition and Technological Development, Universitat Politècnica de València, 46100 Valencia, Spain

[4]IDAL, Intelligent Data Analysis Laboratory, ETSE, Universitat de València, 46100 Valencia, Spain

Corresponding author email: manuel.pastor@upf.edu

Table S1: ML models, the grid of hyperparameters, the best parameters and the best score for regression models built for predicting pIC_{50} of each inhibited transporter, computed on the training series: **A)** P-gp **B)** BCRP **C)** OATP1B1 **D)** OATP1B3 **E)** MRP4 **F)** MRP2 **G)** BSEP **H)** MRP3. The colour green shows the selected algorithm.

A) P-gp

ML models	Grid of hyperparameters	Best parameters	Best MAE score

**XGB**	Colsample_bytree=[0.5-1], max_depth=[None-8], min_child_weight=[1-6], n_estimators=[16-100]	Colsample_bytree=0.5, max_depth=8, min_child_weight=2, n_estimators=100	1.01
**RF**	n_estimators=[16-100], max_depth=[None-8],criterion=[None,mse], min_samples_split=[2-5]	N_estimators=16, criterion=mse, max_depth=2, min_samples_split=2	0.95
**KNN**	N_neighbors=[1-31]	N_neighbors=1	1.21
**SVM**	Kernel=[linear,rbf],C=[1,10],gamma=[0.001,0.0001]	C=10, gamma=0.001, kernel=linear	1.37

B) BCRP

ML models	Grid of hyperparameters	Best parameters	Best MAE score
**XGB**	Colsample_bytree=[0.5-1], max_depth=[None-8], min_child_weight=[1-6], n_estimators=[16-100]	Colsample_bytree=0.5, max_depth=4, min_child_weight=1, n_estimators=100	0.57
**RF**	n_estimators=[16-100], max_depth=[None-8],criterion=[None,mse], min_samples_split=[2-5]	N_estimators=16, criterion=mse, max_depth=None, min_samples_split=2	0.53
**KNN**	N_neighbors=[1-31]	N_neighbors=1	0.67
**SVM**	Kernel=[linear,rbf],C=[1,10],gamma=[0.001,0.0001]	C=10, gamma=0.001, kernel=linear	0.60

C) OATP1B1

ML models	Grid of hyperparameters	Best parameters	Best MAE score

**XGB**	Colsample_bytree=[0.5-1], max_depth=[None-8], min_child_weight=[1-6], n_estimators=[16-100]	Colsample_bytree=0.5, max_depth=None, min_child_weight=2, n_estimators=100	0.41
**RF**	n_estimators=[16-100], max_depth=[None-8],criterion=[None,mse], min_samples_split=[2-5]	N_estimators=16, criterion=mse, max_depth=2, min_samples_split=5	0.38
**KNN**	N_neighbors=[1-31]	N_neighbors=30	0.51
**SVM**	Kernel=[linear,rbf],C=[1,10],gamma=[0.001,0.0001]	C=1, gamma=0.0001, kernel=rbf	0.50

D) OATP1B3

**ML models**	**Grid of hyperparameters**	**Best parameters**	**Best MAE score**
**XGB**	Colsample_bytree=[0.5-1], max_depth=[None-8], min_child_weight=[1-6], n_estimators=[16-100]	Colsample_bytree=1, max_depth=8, min_child_weight=1, n_estimators=80	0.84
**RF**	n_estimators=[16-100], max_depth=[None-8],criterion=[None,mse], min_samples_split=[2-5]	N_estimators=16, criterion=mse, max_depth=None, min_samples_split=3	0.74
**KNN**	N_neighbors=[1-31]	N_neighbors=1	0.79
**SVM**	Kernel=[linear,rbf],C=[1,10],gamma=[0.001,0.0001]	C=1, gamma=0.0001, kernel=rbf	0.70

E) MRP4

**ML models**	**Grid of hyperparameters**	**Best parameters**	**Best MAE score**

**XGB**	Colsample_bytree=[0.5-1], max_depth=[None-8], min_child_weight=[1-6], n_estimators=[16-100]	Colsample_bytree=1, max_depth=2, min_child_weight=2, n_estimators=100	0.48
**RF**	n_estimators=[16-100], max_depth=[None-8],criterion=[None,mse], min_samples_split=[2-5]	N_estimators=80, criterion=mse, max_depth=None, min_samples_split=3	0.44
**KNN**	N_neighbors=[1-31]	N_neighbors=2	0.52
**SVM**	Kernel=[linear,rbf],C=[1,10],gamma=[0.001,0.0001]	C=1, gamma=0.001, kernel=linear	0.48

F) MRP2

ML models	Grid of hyperparameters	Best parameters	Best MAE score
**XGB**	Colsample_bytree=[0.5-1], max_depth=[None-8], min_child_weight=[1-6], n_estimators=[16-100]	Colsample_bytree=0.5, max_depth=8, min_child_weight=2, n_estimators=100	0.34
**RF**	n_estimators=[16-100], max_depth=[None-8],criterion=[None,mse], min_samples_split=[2-5]	N_estimators=16, criterion=mse, max_depth=None, min_samples_split=3	0.32
**KNN**	N_neighbors=[1-31]	N_neighbors=26	0.38
**SVM**	Kernel=[linear,rbf],C=[1,10],gamma=[0.001,0.0001]	C=1, gamma=0.0001, kernel=rbf	0.36

G) BSEP

ML models	Grid of hyperparameters	Best parameters	Best MAE score

XGB	Colsample_bytree=[0.5-1], max_depth=[None-8], min_child_weight=[1-6], n_estimators=[16-100]	Colsample_bytree=1, max_depth=2, min_child_weight=2, n_estimators=16	0.34
RF	n_estimators=[16-100], max_depth=[None-8],criterion=[None,mse], min_samples_split=[2-5]	N_estimators=16, criterion=mse, max_depth=2, min_samples_split=5	0.35
KNN	N_neighbors=[1-31]	N_neighbors=27	0.37
SVM	Kernel=[linear,rbf],C=[1,10],gamma=[0.001,0.0001]	C=1, gamma=0.0001, kernel=rbf	0.38

H) MRP3

ML models	Grid of hyperparameters	Best parameters	Best MAE score
XGB	Colsample_bytree=[0.5-1], max_depth=[None-8], min_child_weight=[1-6], n_estimators=[16-100]	Colsample_bytree=0.5, max_depth=None, min_child_weight=4, n_estimators=100	0.46
RF	n_estimators=[16-100], max_depth=[None-8],criterion=[None,mse], min_samples_split=[2-5]	N_estimators=16, criterion=mse, max_depth=8, min_samples_split=5	0.38
KNN	N_neighbors=[1-31]	N_neighbors=1	0.47
SVM	Kernel=[linear,rbf],C=[1,10],gamma=[0.001,0.0001]	C=1, gamma=0.001, kernel=linear	0.37

Table S2: ML models, the grid of hyperparameters, the best parameters and the best score for classification direct QSAR models predicting cholestasis: **A)** 20 Repeated 5-fold CV **B)** Similarity 5-fold CV. The colour green shows the selected algorithm.

A) 5-fold CV

	Algorithms	Grid of hyperparameters	Best parameters	Best ROC AUC score
QSAR_model_FP	XGB	min_child_weight=[1-10], gamma=[0.5-5], max_depth=[None-12], subsample=[0.6-1.0]	min_child_weight=1, gamma=0.5, max_depth=2, subsample=1.0	0.61
	RF	N_estimators=[16-300], criterion=[gini,entropy], min_samples_split=[2-5], max_depth=[None-12], class_weight=[None,balanced,[class_0:0-1,class_1:2-3]]	n_estimators=100, criterion=gini, min_samples_split=5, max_depth=4, class_weight=balanced	0.65
	MNB	Alpha=[1e-9-1]	Alpha=1e-9	0.57
	SVM	Kernel=[linear,rbf], gamma=[1e-4-1e-3], class_weight=[None,balanced,[class_0:0-1,class_1:2-3]]	kernel=rbf, gamma=0.001, C=10, class_weight=balanced	0.64
QSAR_model_PC	XGB	min_child_weight=[1-10], gamma=[0.5-5], max_depth=[None-12], subsample=[0.6-1.0]	min_child_weight=1, gamma=1.5, max_depth=None, subsample=0.8	0.60
	RF	N_estimators=[16-300], criterion=[gini,entropy], min_samples_split=[2-5], max_depth=[None-12], class_weight=[None,balanced,[class_0:0-1,class_1:2-3]]	n_estimators=300, criterion=gini, min_samples_split=5, max_depth=4, class_weight=balanced	0.62
	GNB	Var_smoothing=[1e-9-10]	Var_smoothing=9.8e-5	0.52
	SVM	Kernel=[linear,rbf], gamma=[1e-4-1e-3], class_weight=[None,balanced,[class_0:0-1,class_1:2-3]]	kernel=rbf, gamma=0.001, C=10, class_weight=balanced	0.60
Metamodel_pk	Logical_OR	n=[0-15]	n=4.70	0.69
Metamodel_not_pk	Logical_OR	Thres=[0.1-300]	Thres=3.13	0.50

B) Similarity 5-fold CV

	Algorithms	Grid of hyperparameters	Best parameters	Best ROC AUC score
QSAR_model_FP	XGB	min_child_weight=[1-10], gamma=[0.5-5], max_depth=[None-12], subsample=[0.6-1.0]	min_child_weight=1, gamma=0.5, max_depth=8, subsample=1	0.57
	RF	N_estimators=[16-300], criterion=[gini,entropy], min_samples_split=[2-5], max_depth=[None-12], class_weight=[None,balanced,[class_0:0-1,class_1:2-3]]	n_estimators=16, criterion=entropy, min_samples_split=2, max_depth=8, class_weight=balanced	0.54
	MNB	Alpha=[1e-9-1]	Alpha=1e-9	0.53
	SVM	Kernel=[linear,rbf], gamma=[1e-4-1e-3], class_weight=[None,balanced,[class_0:0-1,class_1:2-3]]	kernel=linear, gamma=0.001, C=10, class_weight=None	0.52
QSAR_model_PC	XGB	min_child_weight=[1-10], gamma=[0.5-5], max_depth=[None-12], subsample=[0.6-1.0]	min_child_weight=1, gamma=0.5, max_depth=2, subsample=1	0.57
	RF	N_estimators=[16-300], criterion=[gini,entropy], min_samples_split=[2-5], max_depth=[None-12], class_weight=[None,balanced,[class_0:0-1,class_1:2-3]]	n_estimators=100, criterion=entropy, min_samples_split=2, max_depth=4, class_weight=balanced	0.56
	GNB	Var_smoothing=[1e-9-10]	Var_smoothing=9.2e-5	0.48
	SVM	Kernel=[linear,rbf], gamma=[1e-4-1e-3], class_weight=[None,balanced,[class_0:0-1,class_1:2-3]]	kernel=rbf, gamma=0.001, C=10, class_weight=balanced	0.53
Metamodel_pk	Logical_OR	n=[0-15]	n=5.5	0.67
Metamodel_not_pk	Logical_OR	Thres=[0.1-300]	Thres=1.0	0.52

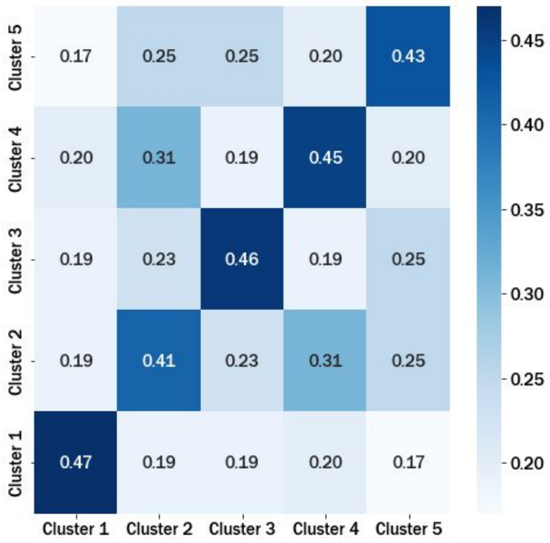

Figure S1: Tanimoto similarity values computed intra and inter-cluster.

Table S3: ML models, the grid of hyperparameters, the best parameters and the best score for classification direct QSAR models predicting cholestasis for the top five ATC codes using a grid search ATC 5-fold CV. The colour green shows the selected algorithm.

	Algorithms	Grid of hyperparameters	Best parameters	Best ROC AUC score
QSAR_model_FP	XGB	min_child_weight=[1-10], gamma=[0.5-5], max_depth=[None-12], subsample=[0.6-1.0]	min_child_weight=1, gamma=0.5, max_depth=4, subsample=0.6	0.62
	RF	N_estimators=[16-300], criterion=[gini,entropy], min_samples_split=[2-5], max_depth=[None-12], class_weight=[None,balanced,[class_0:0-1,class_1:2-3]]	n_estimators=32, criterion=entropy, min_samples_split=2, max_depth=4, class_weight=balanced	0.60
	MNB	Alpha=[1e-9-1]	Alpha=1e-9	0.60
	SVM	Kernel=[linear,rbf], gamma=[1e-4-1e-3], class_weight=[None,balanced,[class_0:0-1,class_1:2-3]]	kernel=linear, gamma=0.001, C=1, class_weight=balanced	0.59
QSAR_model_PC	XGB	min_child_weight=[1-10], gamma=[0.5-5],	min_child_weight=1, gamma=1, max_depth=4, subsample=0.6	0.60

		max_depth=[None-12], subsample=[0.6-1.0]		
	**RF**	N_estimators=[16-300], criterion=[gini,entropy], min_samples_split=[2-5], max_depth=[None-12], class_weight=[None,balanced,[class_0:0-1,class_1:2-3]]	n_estimators=16, criterion=gini, min_samples_split=4, max_depth=None, class_weight=balanced	0.55
	**GNB**	Var_smoothing=[1e-9-10]	Var_smoothing=1.0e-5	0.53
	**SVM**	Kernel=[linear,rbf], gamma=[1e-4-1e-3], class_weight=[None,balanced,[class_0:0-1,class_1:2-3]]	kernel=rbf, gamma=0.001, C=10, class_weight=balanced	0.59
**Metamodel_pk**	**Logical_OR**	n=[0-15]	n=4.7	0.73
**Metamodel_not_pk**	**Logical_OR**	Thres=[0.1-300]	Thres=3.13	0.51

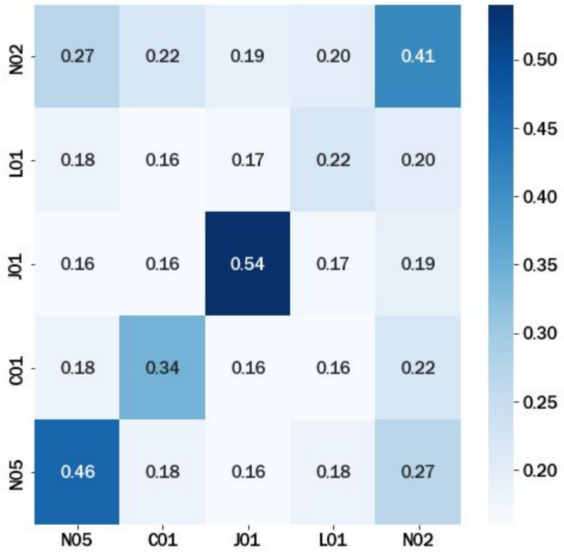

Figure S2: Tanimoto similarity values computed intra and inter-ATC.

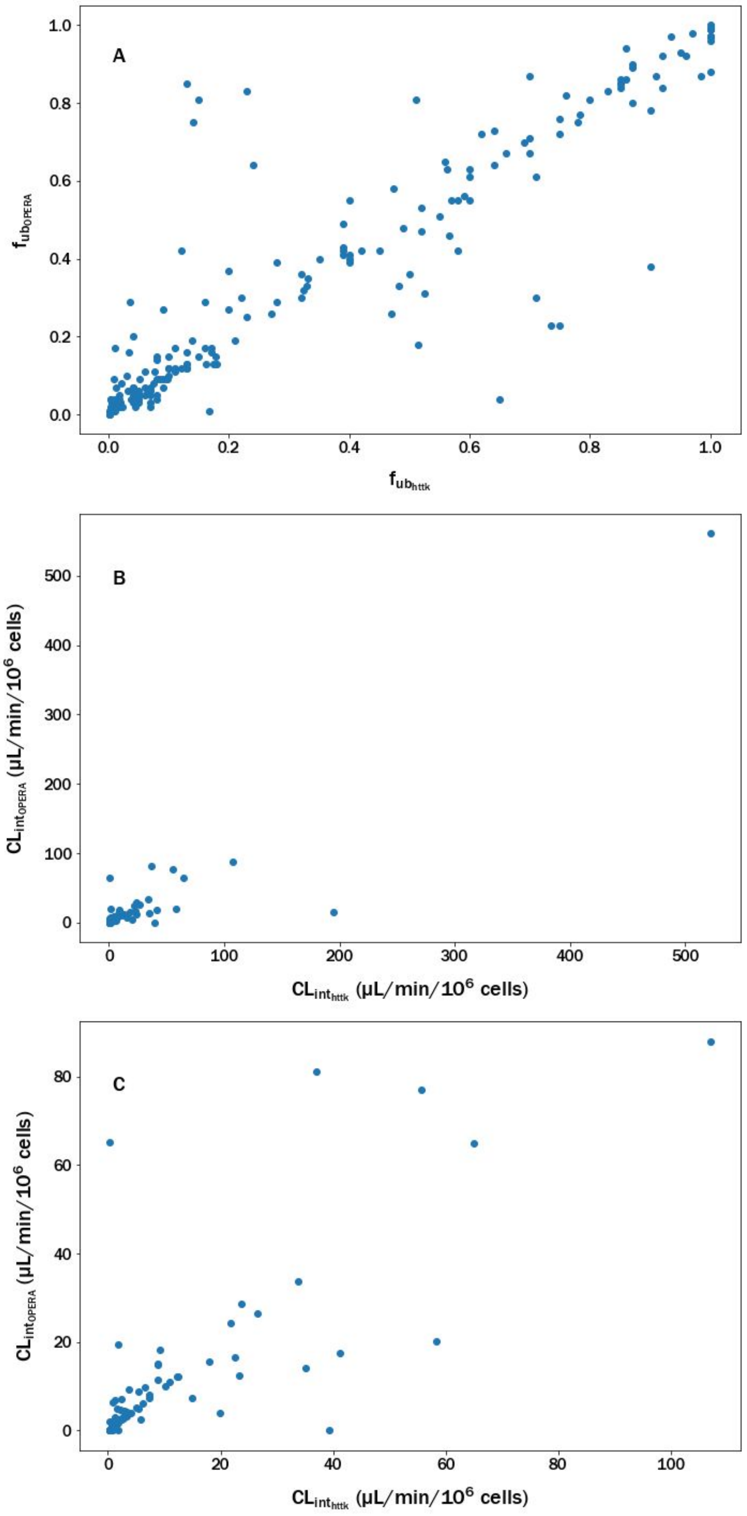

Figure S3: Scatter plots for compounds of cholestasis dataset with experimental values available in httk for f_{ub} (**A**) and Cl_{int} (**B**). The X-axis represents the httk values, while the Y-axis represents the corresponding values predicted by OPERA. (**C**) This is a zoom of S3 removing compounds with CL_{int} >195 μL/min/10^6cells.

Table S4: f_{ub} and Cl_{int} MAEs for compounds with experimental httk activity comparing the predicted values from OPERA, mean, and std of the experimental httk values and the predicted values by OPERA.

Metrics	f_{ub} (#Compounds=205)	CL_{int} (#Compounds=75)
MAE	0.07	8.50
$Mean_{httk}$	0.34	20.73
std_{httk}	0.34	64.96
$Mean_{OPERA}$	0.35	19.27
std_{OPERA}	0.33	66.15

Table S5: Selected metrics per fold for each model of the Similarity 5-fold CV with the similarity computed intra-cluster.

		Metamodel_pk	Metamodel_not_pk	QSAR_model_FP	QSAR_model_PC	Cluster_validation
Sensitivity	Fold_1	0.83	0.17	0.17	0.33	1
	Fold_2	0.78	0.02	0.09	0.22	2
	Fold_3	0.88	0.04	0.17	0.46	3
	Fold_4	0.87	0.00	0.20	0.13	4
	Fold_5	0.71	0.07	0.36	0.07	5
Specificity	Fold_1	0.64	0.96	0.93	0.91	1
	Fold_2	0.61	0.95	0.97	0.89	2
	Fold_3	0.35	1.00	0.89	0.84	3
	Fold_4	0.72	1.00	0.97	0.91	4
	Fold_5	0.35	0.98	0.92	0.95	5
MCC	Fold_1	0.31	0.17	0.12	0.24	1
	Fold_2	0.37	-0.08	0.15	0.14	2
	Fold_3	0.25	0.16	0.08	0.32	3
	Fold_4	0.47	0.00	0.27	0.05	4
	Fold_5	0.06	0.11	0.35	0.04	5
AUC	Fold_1	0.74	0.56	0.55	0.62	1
	Fold_2	0.70	0.48	0.53	0.55	2
	Fold_3	0.61	0.52	0.53	0.65	3
	Fold_4	0.79	0.50	0.58	0.52	4
	Fold_5	0.53	0.52	0.64	0.51	5
Ac	Fold_1	0.67	0.86	0.84	0.84	1
	Fold_2	0.67	0.66	0.70	0.68	2

	Fold_3	0.56	0.62	0.61	0.69	3
	Fold_4	0.75	0.81	0.82	0.76	4
	Fold_5	0.44	0.74	0.78	0.72	5

Table S6: Mean and std of the Sensitivity (S), specificity (SP), AUC, MCC, and Accuracy (A) for each model in the ATC 5-fold CV.

	S	S_std	SP	SP_std	AUC	AUC_std	MCC	MCC_std	A	A_std
Metamodel_pk	0.92	0.11	0.54	0.25	0.73	0.12	0.39	0.13	0.68	0.07
Metamodel_not_pk	1.00	0.00	0.01	0.03	0.51	0.01	0.03	0.05	0.29	0.15
QSAR_model_FP	0.33	0.21	0.85	0.18	0.59	0.11	0.20	0.25	0.70	0.16
QSAR_model_PC	0.07	0.13	0.87	0.10	0.47	0.07	-0.07	0.14	0.63	0.10

Table S7: Selected metrics per fold for each model of the ATC 5-fold CV with the similarity computed for each fold.

		Metamodel_pk	Metamodel_not_pk	QSAR_model_FP	QSAR_model_PC	ATC_validation
Sensitivity	Fold_1	1.00	1.00	0.15	0.00	J01
	Fold_2	0.75	1.00	0.5	0.00	N05
	Fold_3	0.83	1.00	0.5	0.33	L01
	Fold_4	1.00	1.00	0.00	0.00	C01
	Fold_5	1.00	1.00	0.5	0.00	N02
Specificity	Fold_1	0.06	0.00	0.94	1.00	J01
	Fold_2	0.60	0.00	0.96	0.96	N05
	Fold_3	0.69	0.06	0.50	0.81	L01
	Fold_4	0.73	0.00	0.93	0.73	C01
	Fold_5	0.64	0.00	0.93	0.86	N02
MCC	Fold_1	0.18	0.00	0.15	0.00	J01
	Fold_2	0.33	0.00	0.55	-0.12	N05
	Fold_3	0.47	0.13	0.00	0.15	L01
	Fold_4	0.56	0.00	-0.11	-0.24	C01
	Fold_5	0.43	0.00	0.43	-0.14	N02
AUC	Fold_1	0.53	0.50	0.55	0.50	J01
	Fold_2	0.68	0.50	0.73	0.48	N05
	Fold_3	0.76	0.53	0.5	0.57	L01

	**Fold_4**	0.87	0.50	0.47	0.37	C01
	**Fold_5**	0.82	0.50	0.71	0.43	N02
**Accuracy**	**Fold_1**	0.57	0.54	0.51	0.46	J01
	**Fold_2**	0.65	0.32	0.81	0.65	N05
	**Fold_3**	0.73	0.32	0.50	0.68	L01
	**Fold_4**	0.78	0.17	0.78	0.61	C01
	**Fold_5**	0.69	0.12	0.88	0.75	N02
